# Advancing new metrics for wildfire smoke exposure: case study in Alaska to bridge public health, climate adaptation, and fire management

**DOI:** 10.1088/1748-9326/adeff6

**Published:** 2025-07-25

**Authors:** Micah B Hahn, Nelsha R Athauda, Zhiwei Dong, Melissa Bradley, Jingqiu Mao, Loretta J Mickley

**Affiliations:** 1 Institute for Circumpolar Health Studies, University of Alaska Anchorage, Anchorage, AK, United States of America; 2 Geophysical Institute and Department of Chemistry and Biochemistry, University of Alaska Fairbanks, Fairbanks, AK, United States of America; 3 John A. Paulson School of Engineering and Applied Sciences, Harvard University, Cambridge, MA, United States of America

**Keywords:** wildfire smoke, human health, environmental justice, exposure metrics, Alaska

## Abstract

Wildfire activity is increasing globally due to climate change, with implications for air quality and public health. Fine particulate matter (PM_2.5_) from wildfire smoke contributes to cardiorespiratory morbidity and mortality, adverse birth outcomes, mental health stressors, and disruptions to food security and traditional livelihoods. However, quantifying health risks remains difficult due to sparse monitoring, challenges in isolating wildfire-specific pollution, and limited long-term exposure assessments. We developed a historical air quality dataset for Alaska using a hybrid approach that integrates GEOS-Chem atmospheric modeling with ground-based data to estimate daily wildfire-attributable PM_2.5_ at a 0.625° × 0.5° resolution from 2003 to 2020. We aggregated these estimates by census tract and derived metrics to quantify long-term wildfire smoke exposure, then combined these estimates with social vulnerability data to identify populations disproportionately affected. Alaskans experienced an average of 3.5 million person-days of moderate and >800 000 person-days of dense smoke exposure annually. In years when over 2 million acres burned, 86%–98% of census tracts recorded at least 1 d of moderate smoke, and up to 73% experienced dense smoke. Northern Interior Alaska had over 300 cumulative days of poor air quality (∼10% of summer days) over the 18 year period, with smoke waves lasting as long as 43 d. Tracts identified as having high smoke exposure and high smoke vulnerability were generally in rural Interior Alaska; however, urban tracts in Interior and Southcentral were also identified. High-exposure census tracts had statistically greater proportions of housing cost-burdened residents and women of childbearing age. This study highlights the need to move beyond traditional fire metrics and adopt measures that better capture the full scope of human exposure. Our approach provides a framework for assessing health risks and integrating public health into climate adaptation and fire management especially in wildfire-prone regions where observations are sparse.

## 1. Introduction

In recent decades, wildfires have increased in frequency and severity in many regions around the world, and the weather conditions associated with wildfire risk (e.g. high temperatures and low humidity) are expected to intensify due to climate change [1, 2]. In the western US and Alaska, these trends have already emerged [3–7], with some of the worst fire seasons on record occurring since 2020 in these regions [8–10]. In addition to the direct impacts of wildfires on infrastructure, natural resources, and community safety, wildfire smoke has become a major source of air pollution across Alaska and the contiguous US, with significant and growing impacts on public health [11, 12]. Acute wildfire smoke exposure is consistently linked with respiratory disease, and to a lesser extent, cardiovascular events [13, 14], with evidence of impacts on birth outcomes [15], skin diseases [16], eye conditions [17], and cancer [18, 19]. Recent studies also highlight mental health impacts, such as anxiety and depression [20–22], and there is growing attention to wildfire smoke as an environmental justice issue due to inequities in exposure and recovery resources [23–28]. These disparities have driven calls for equity-focused wildfire and smoke management strategies that address immediate health risks while fostering systemic changes that transform the way we live with fire [23, 29].

In Alaska, a rapidly changing environment is driving longer fire seasons, more frequent large wildfires, and fires in regions historically unaffected [30]. Wildfire smoke has been linked to an increased risk of acute cardiorespiratory events in urban Alaska [31], and wildfires have cascading impacts on mental health [22] and subsistence food resources critical to Alaska’s rural and Tribal communities [32, 33]. Understanding the health impacts of wildfire smoke and exposure disparities requires long-term smoke data across time and location. However, Alaska’s vast size (land area equivalent to one-fifth of the contiguous US) and sparse air quality monitoring network leaves many communities without historical data [34]. Before 2020, only the state’s three major population centers had air quality monitors [35], and while recent expansions include low-cost and non-regulatory sensors, additional methods are needed to address gaps in rural regions.

An additional challenge for estimating wildfire smoke exposure is distinguishing between particulate matter emitted by wildfires versus other ambient sources. Health studies generally focus on fine particulate matter less than 2.5 microns in diameter (PM_2.5_) [36, 37]. A recent assessment applied a wildfire emission inventory to the Hybrid Single-Particle Lagrangian Integrated Trajectory (HYSPLIT) model to estimate daily smoke PM_2.5_ in Alaska between 2001 and 2015; results showed some discrepancies with the observed data, especially during extreme smoke events [38]. Woo *et al* estimated smoke PM_2.5_ for Alaska between 1997 and 2001, but the grid cell size of their estimates (4° × 5°) precludes application at the community-level [39]. Several studies have blended approaches to estimate smoke PM_2.5_ using a combination of data from ground-based sensors, satellites, and chemical transport models [40–43]. We previously combined satellite information with ground monitor data to estimate smoke PM_2.5_ in urban Alaska [31], and more recently, we estimated surface PM_2.5_ during wildfire seasons in selected years across Alaska [34]. However, the latter approach is limited by satellite aerosol optical depth availability and uncertainties in the vertical profiles of smoke. To generate state-wide estimates over the past two decades, it was necessary to incorporate modeled data.

Despite growing evidence linking wildfire smoke to health impacts, significant knowledge gaps remain. Most epidemiologic studies treat wildfire smoke exposure as an acute event, overlooking its cyclical and sometimes prolonged nature. For instance, there are limited data on the health effects of repeated short-term exposures or prolonged smoke events lasting several weeks. In studies examining long-term smoke exposure, researchers often use average exposure over time [19, 44, 45], which can mask spikes in smoke PM_2.5_. To more accurately characterize wildfire smoke exposure, we need methods that capture intra- and inter-seasonal variability and long-term smoke. For example, Heft-Neal *et al* utilized a metric to account for the number of days of exposure and the concentration of smoke PM_2.5_ over a gestational period in their study on birth outcomes [46], and Casey *et al* proposed several smoke metrics to account for duration, frequency, and concentration of smoke exposure over an 8 year study period [25]. Beyond exposure, population sensitivity and response capacity shape vulnerability to wildfire smoke. Identifying highly vulnerable communities supports mitigation and adaptation efforts while helping policymakers and agencies allocate resources and coordinate public health responses [41, 47, 48].

In this study, we address these key challenges: (1) estimating air quality in areas with limited monitoring data, (2) distinguishing wildfire-specific particulate matter from other sources when combining modeled and observed air pollution data, (3) assessing seasonal and long-term exposure to wildfire smoke, and (4) identifying populations disproportionately affected by wildfire smoke events. We used a hybrid approach that integrated outputs from the 3D GEOS-Chem atmospheric transport model with ground-based monitoring data to estimate daily smoke PM_2.5_ concentrations on a 0.625° × 0.5° grid across Alaska from 2003 to 2020. We aggregated these estimates to the census tract level to create smoke metrics, and then combined these with indicators of social vulnerability to categorize tracts based on exposure and disadvantage levels.

## 2. Methods

### 2.1. Development of historical smoke PM_2.5_ dataset for Alaska

#### 2.1.1. Air quality monitor data

Two sets of air quality monitor data were used in this study. PM_2.5_ data were obtained from ground-monitoring sensors maintained by the Alaska Department of Environmental Conservation (AK DEC) for Environmental Protection Agency (EPA) regulatory monitoring. We utilized daily average data (based on hourly samples) from six AK DEC sensors including three in Fairbanks, two in Anchorage, and one in Juneau. Daily data were excluded if fewer than 75% of hourly measurements were taken or if the recorded PM_2.5_ concentration was implausible (e.g. ⩽0 *μ*g m^−3^).

PurpleAir is a low-cost sensor that has been used since 2019 to improve the spatial coverage of real-time air quality information in Alaska. We utilized the available public data from PurpleAir sensors which included information from 2019 (20 sensors) and 2020 (56 sensors). Sensors were located primarily around Fairbanks and Anchorage, with additional sensors in communities in Interior, Southwest, Southcentral, and Southeast Alaska. We utilized hourly observations to calculate daily average PM_2.5_. Daily data were excluded if fewer than 75% of hourly measurements were available. Data from PurpleAir monitors require calibration, and we utilized a calibration equation (equation (1)) applicable to the US [49],

\begin{align*}{\text{P}}{{\text{M}}_{{\text{2}}{\text{.5}}}} = 0.524*{\text{PurpleAi}}{{\text{r}}_{cf1}} - { }0.0862*{\text{RH}} + 5.75\end{align*}

where PM_2.5_ is concentration in *μ*g m^−3^; PurpleAir*_cf*1 represents the average PM_2.5_ records from two higher correction factor (*cf*1) channels on the sensor; and RH represents relative humidity, also collected by the sensor. The calibrated PurpleAir PM_2.5_ had *R*^2^ as high as 0.93, in good agreement with nearby AK DEC PM_2.5_ (supplemental figure 1).

#### 2.1.2. GEOS-Chem chemical transport model

GEOS-Chem is a 3D chemical transport model that has been widely used in wildfire smoke transport [42, 50] and health effects studies [51, 52]. Here, we use GEOS-Chem (version 12.7.2) to simulate daily surface PM_2.5_ emitted from wildfires in Alaska for the period of May–September, 2003–2020. Meteorological fields from the Modern Era Retrospective Analysis for Research and Applications, Version 2 were used to drive GEOS-Chem, and daily and 3 h fractions from the Global Fire Emissions Database, Version 4 (GFED4) [53] were used to represent diurnally varying wildfire emissions [54]. Wildfire emissions are injected into the surface layer and redistributed in the planetary boundary layer (PBL) based on a non-local mixing scheme [55]. Dry deposition follows the resistance-in-series scheme by Wesely [56]. Wet deposition includes scavenging in convective updrafts, mid-level entrainment and detrainment, and rainout and washout from convective anvils and large-scale precipitation [57]. To isolate smoke PM_2.5_, we performed two simulations: one nested simulation around the Alaska domain (127.5–172.5°W, 47.5–77.5°N) at 0.5° × 0.625° resolution with GFED4 emissions to estimate total PM_2.5_ and another global simulation at 2° × 2.5° resolution without GFED4 emissions to estimate non-smoke PM_2.5_. The non-smoke output in Alaska was interpolated to 0.5° × 0.625°. To assess the validity of the interpolation, we conducted a separate nested simulation for 2014 without GFED4 emissions and compared its background PM_2.5_ results with the interpolated coarse-resolution fields. The spatial *R*^2^ between the two datasets is high (0.93–0.98 across summer months), and their distributions are highly similar, with little differences in median values (supplemental figure 2). This confirms that the results interpolated from the coarser resolution model sufficiently captured the magnitude and variability of background (non-wildfire) PM_2.5_ levels, which are typically low in summer months in Alaska, when anthropogenic emissions from traffic, domestic heating, and industry are relatively small.

#### 2.1.3. PM_2.5_ data blending and estimation of smoke PM_2.5_

To improve the accuracy of GEOS-Chem PM_2.5_ simulations, we calibrated the modeled results with air quality data from AK DEC and PurpleAir sensors. Since sensor data reflect total PM_2.5_, we blended these data with GEOS-Chem total PM_2.5_. We used quantile mapping, a method that aligns the distributions of the modeled and observed datasets by establishing relationships within quantile ranges [58]. For extremely high modeled values, adjustments were capped at the observed 100th quantile to avoid bias from outliers. The overestimation of GEOS-Chem PM_2.5_ simulations during high wildfire activity years was significantly reduced after the adjustment (supplemental figure 3). Leave-one-out cross-validation showed that our quantile-mapping method largely reduced RMSE and increased *R*^2^ for most locations (supplemental figure 4). A 20-fold cross validation using PurpleAir observations in summer of 2019 showed *R*^2^ of 0.54 and a subsequent cross validation incorporating both PurpleAir and DEC observations from 8 years, including four high wildfire activity years (2004, 2005, 2009 and 2019) and four low activity years (2007, 2008, 2014 and 2020), showed an overall *R*^2^ of 0.60 (supplemental figure 5).

Next, we replaced GEOS-Chem PM_2.5_ estimates in grid cells containing sensors with the observed values from those sensors (or average value if a grid cell contained multiple sensors). Biases between the quantile-mapped estimates and observations in these grid cells were then propagated to the surrounding 2–3 cells using the successive correction method (SCM), which adjusts model values based on distance from sensor-equipped cells [59, 60]. Since the bias is largely reduced by the quantile-mapping step, the SCM correction has only a minor impact on the final values and nearby grid cells. No significant gradients were observed near these sensors after the SCM calibration (supplemental figure 6). To estimate the daily fractional contribution of smoke PM_2.5_ to total PM_2.5_ in each grid cell, we then subtracted the ratio of non-smoke PM_2.5_ to total PM_2.5_ from one. In the last step, we multiplied the calibrated total PM_2.5_ output by this fraction to obtain the final calibrated smoke PM_2.5_ in that grid cell. We further evaluated our smoke PM_2_._5_ estimates using the hazard mapping system (HMS) product, which may indicate surface smoke in Alaska [61]. Regions with high smoke contributions in our results generally fall within HMS-defined smoke areas on heavily smoky days (supplemental figure 7).

### 2.2. Exposure assessment

#### 2.2.1. Data sources and development of wildfire smoke PM_2.5_ metrics

To estimate census tract level exposure to wildfire smoke between 2003 and 2020, we combined the final daily smoke PM_2.5_ dataset with Alaska census tract boundaries from the Alaska Department of Labor and Workforce Development [62] using zonal statistics. We calculated average daily smoke PM_2.5_ using a weighted mean, with grid cell values weighted by the proportion of each cell within the tract. Because census tracts are based on population, the geographic size of Alaska census tracts is highly variable (range: 0.65–224 641.5 km^2^), but on average, Alaska census tracts are 92 times larger than in the contiguous US (AK: 8590 km^2^; US: 93 km^2^). At the grid resolution used for the GEOS-Chem output in this study (0.5° × 0.625°), 62.7% (*n* = 111) of tracts overlap with 1–2 grid cells, while one of the larger rural tracts overlaps with ∼170 grid cells (supplemental figure 8). We obtained population estimates for each tract from the 2020 US census population estimates included with the census tract boundary shapefile.

We calculated metrics that collectively measured the frequency, duration, and concentration [63, 64] of long-term exposure to smoke PM_2.5_, adapting and expanding on a framework described by Casey *et al* (supplemental table 1) [25]. Given the uncertainties in the health effects of smoke exposure, we have constructed an array of metrics to capture a range of possible health responses. We first applied EPA air quality index (AQI) standards [65] to categorize daily smoke PM_2.5_ concentration: ‘moderate’ smoke corresponded to the ‘Moderate’ AQI range (9.1–35.4 *μ*g m^−3^), while ‘dense’ smoke included values in the ‘Unhealthy for Sensitive Groups’ range and higher (⩾35.5 *μ*g m^−3^), encompassing the ‘Unhealthy,’ ‘Very Unhealthy,’ and ‘Hazardous’ categories. Given recent evidence of the health impacts of exposure to even low levels of PM_2.5_ [66], we defined days with poor air quality as those days with moderate or dense smoke PM_2.5_.

We calculated weekly and annual person-days of exposure to moderate and dense wildfire smoke by multiplying the population of each census tract by the number of days that tract experienced smoke PM_2.5_ within each exposure category during the specified time period. To contextualize wildfire activity, we compared these exposure values with the total acres burned in Alaska during the same time frame. We also compared person-days of smoke exposure by census tract for 2003–2011 and 2012–2020 to assess trends over time.

We calculated the mean daily smoke PM_2.5_ annually and during the wildfire season (May 1–September 30), setting smoke PM_2.5_ to zero outside this period. To capture peak exposures, we computed the mean daily smoke PM_2.5_ for the 10 d each year with the highest PM_2.5_ values within each census tract. We also calculated the number of days each tract experienced moderate or dense smoke, as well as the number of days with dense smoke only. Finally, we identified and quantified smoke waves, defined as two more consecutive days with moderate or dense smoke, and we determined the longest smoke wave in each tract. All metrics were calculated annually and summarized for the 2003–2020 period. We utilized the boundaries of Alaska wildland fire history from the same time period from the National Interagency Fire Center to see the extent of wildfire smoke impact relative to fire locations [67]. Correlations of the wildfire smoke metrics were assessed using pairwise Pearson statistics.

#### 2.2.2. Identifying populations disproportionately affected by wildfire smoke

We created a wildfire smoke social vulnerability index (WSSVI) focused on two themes: (1) health sensitivity to wildfire smoke and (2) adaptive capacity. Here, adaptive capacity is defined as the ability to respond or adapt to wildfire smoke events. To derive these indices, we relied on three secondary data sources: (1) US Census Bureau American Community Survey, (2) Centers for Disease Control and Prevention (CDC) Social Vulnerability Index, and (3) CDC PLACES [68–70]. Information on data sources and definitions for specific variables is available in supplemental table 2.

We measured health sensitivity based on the proportion of individuals that are highly sensitive to wildfire smoke within a tract including: individuals aged 17 and younger; individuals aged 65 and older; those with asthma, chronic obstructive pulmonary disease, or cardiovascular disease; women of childbearing age; and outdoor workers. The selection of these seven variables was based on EPA guidance on populations who experience greater adverse health effects from wildfire smoke exposure [71]. We used the proportion of women of childbearing age as a proxy for pregnant people due to a lack of readily available tract-level information.

We measured adaptive capacity by assessing variables that may reflect the ability of populations within a census tract to respond or adapt to wildfire smoke events. We included three demographic variables: civilians with disabilities, single-parent households, and individuals with limited English proficiency [71–73]. To capture socioeconomic information, we included five variables: individuals below 150% of the federal poverty limit, housing cost-burdened households with an annual income under $75 000, unemployed individuals over 16 years of age, the uninsured, and people over 25 years of age without a high school diploma [24, 48, 74].

To calculate the WSSVI, census tracts were ordered and ranked from lowest proportion to highest proportion for each health sensitivity and adaptive capacity indicator. Next, we summed the indicator rankings within the health sensitivity and adaptive capacity themes and then ranked those values, producing a ranking for each theme. The health sensitivity and adaptive capacity ranks were summed, and then re-ranked to produce the WSSVI for each census tract [75].

To identify census tracts with both high smoke exposure (based on the percentile rank of the number of days a tract experienced moderate or dense smoke) and high smoke vulnerability (based on WSSVI), we visualized this combined burden using both a bivariate map and a quadrant map. Finally, we categorized census tracts by ‘high’ (top 50%) and ‘low’ (bottom 50%) smoke exposure and compared the distribution of health sensitivity and adaptive capacity variables within each group to identify major populations of concern, conducting t-tests between groups to determine statistical significance (*a =*0.05).

All analyses were performed using R (version 2024.12.1) [76] Zonal statistics were calculated using the *exactextractr* data package and *sf* package [77, 78]. We obtained US census data via the census API using *tidycensus* [79]. Scripts for the exposure assessment are available in GitHub [80].

## 3. Results

### 3.1. Wildfire smoke PM_2.5_ metrics

**Figure 1.**
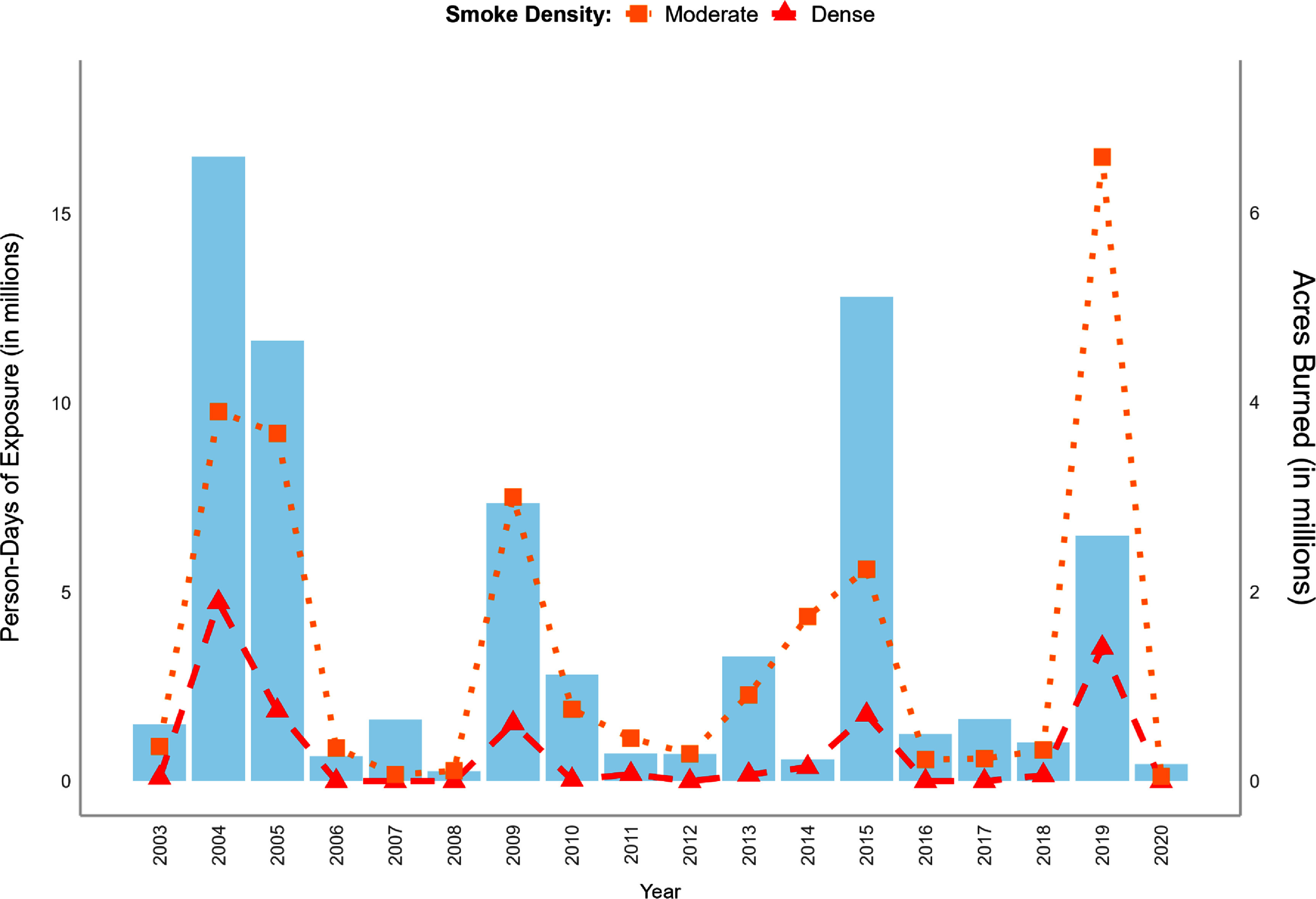
Annual person-days of exposure to wildfire-specific PM_2.5_ (red and orange lines, left hand axis) and acres burned (blue bars, right hand axis) in Alaska, for May–September, 2003–2020. The orange line represents person-days of exposure to moderate smoke, with smoke concentrations of 9.1–35.4 *μ*g m^−3^; the red line represents person-days of exposure to dense smoke, with smoke concentrations of ⩾35.5 *μ*g m^−3^.

Between 2003 and 2020, Alaskan residents experienced nearly 77.8 million person-days of exposure to poor air quality due to wildfire smoke (figure 1). Moderate smoke was present for over 63.3 million person-days, and dense smoke accounted for over 14.4 million person-days. Residents were affected by smoke every year in the study period, and on average, Alaskans experienced 3.5 million person-days of moderate and >800 000 person-days of dense smoke exposure annually. Notably, person-days were not correlated with the total acres burned, a common measure of wildfire season severity. In 2019, despite being the fifth largest wildfire season with nearly 2.5 million acres burned, person-days—which accounts for fire location, smoke transport, and the population exposed—were the highest of the study period, highlighting the substantial population health impact of that season. Similarly, 2014 was a year of relatively low wildfire activity (∼234 000 acres burned), but had the sixth highest number of smoke person-days. Comparing smoke person-days by census tract between 2003–2011 and 2012–2020, nearly two-thirds of tracts (63.3%, *n* = 112) experienced an increase in exposure to moderate smoke, and nearly half (46.3%, *n* = 82) experienced an increase in exposure to dense smoke.

**Figure 2.**
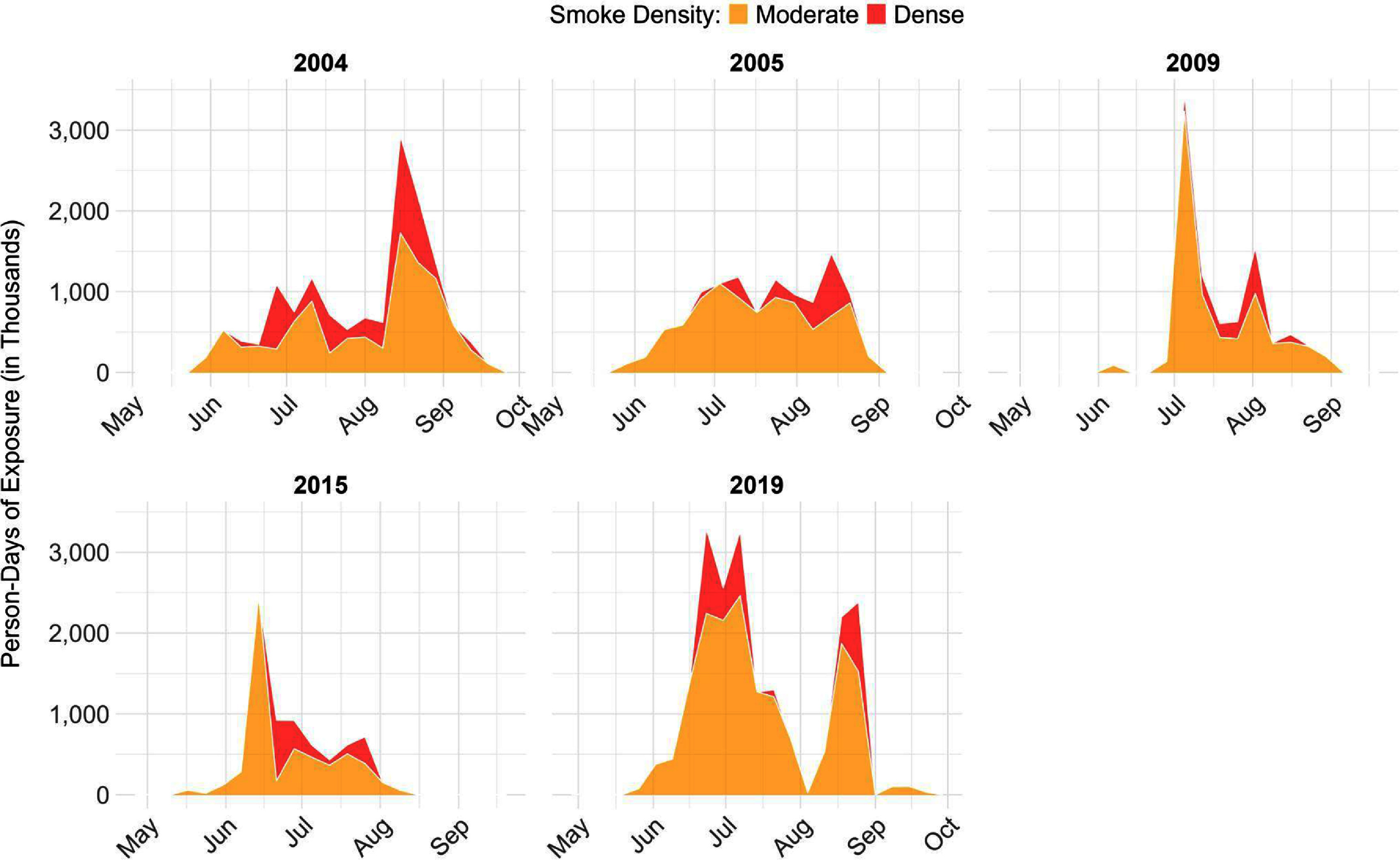
Weekly person-days of exposure to wildfire-specific PM_2.5_ in high-fire years (>2 million acres burned) in Alaska, for May–September, 2003–2020. The stacked contours represent moderate smoke exposure in orange (smoke concentrations of 9.1–35.4 *μ*g m^−3^) and dense smoke exposure in red (smoke concentrations of ⩾35.5 *μ*g m^−3^).

Focusing on the 5 years of highest wildfire activity when over 2 million acres burned (2004, 2005, 2009, 2015, and 2019), we calculated substantial variation in the temporal patterns of smoke across the wildfire season (figure 2). Smoke exposure during these seasons generally began in late May or early June and continued through August or early to mid-September. However, in 2019, smoke exposure occurred between the first week of May and final week of September, marking the longest uninterrupted exposure period and also the highest weekly average for person-days of smoke exposure for the study period (384 thousand week^−1^ compared to 83 thousand week^−1^ averaged across 2003–2020). Most years had at least one substantial peak in smoke exposure, although the timing of the exposure varied. The year 2019 had two peaks. Smoke was a continuous concern in all high-fire years, but it was more periodic in years with lower fire activity (supplemental figure 9).

**Figure 3.**
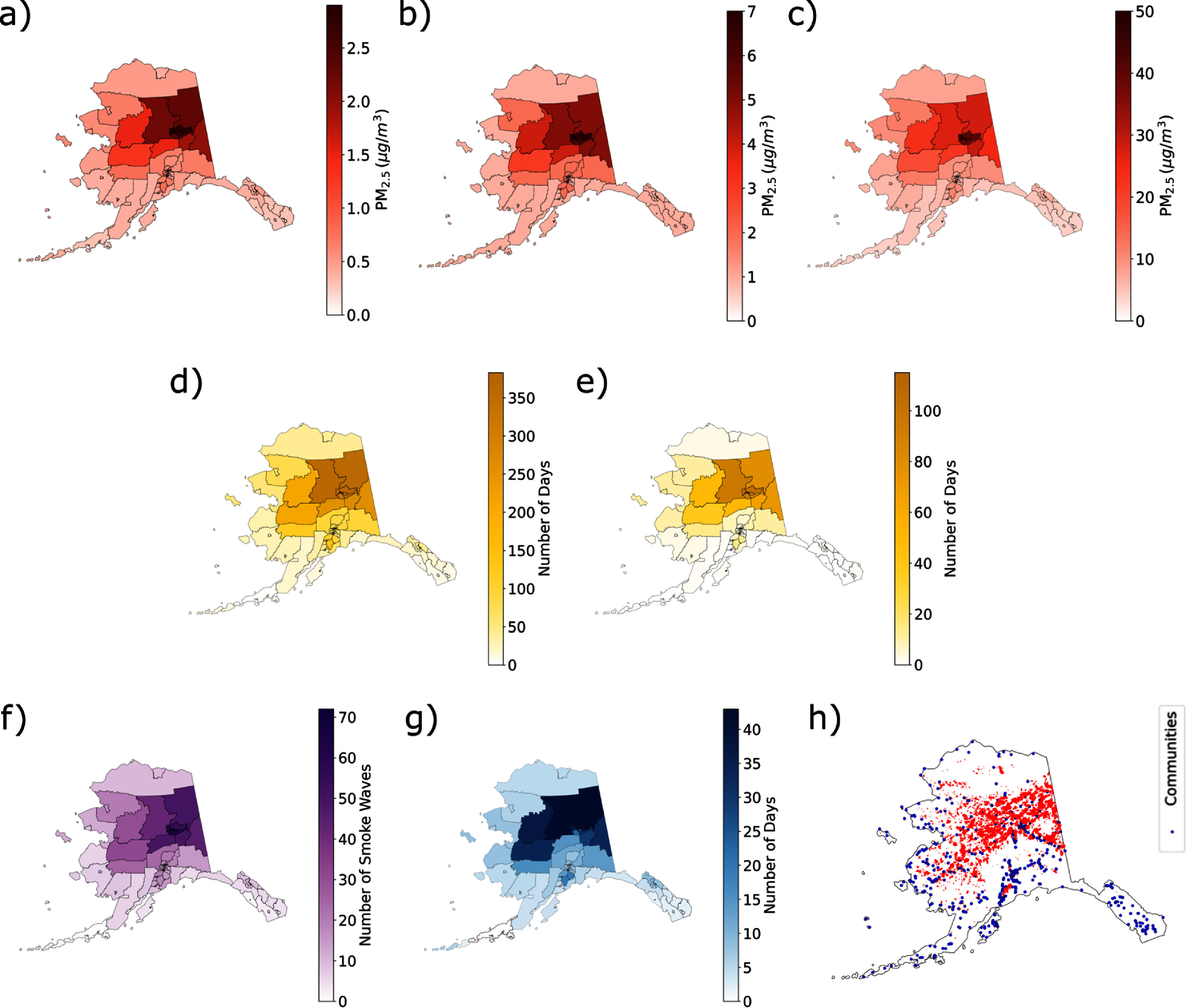
Wildfire smoke metrics for Alaska census tracts, 2003–2020. (a) Annual mean daily smoke PM_2.5_; (b) mean daily smoke PM_2.5_ during wildfire season (May–September); (c) mean smoke PM_2.5_ on ten smokiest days in each tract during wildfire season; (d) total number of days with moderate or dense smoke (⩾9.1 *μ*g m^−3^); (e) total number of days with dense smoke (⩾35.5 *μ*g m^−3^); (f) number of smoke waves (⩾2 consecutive days with moderate or dense smoke); (g) longest smoke waves (in days); (h) point locations of all incorporated and unincorporated Alaska communities (blue) overlaid with cumulative wildfire perimeters from 2003 to 2020 (red).

Between 2003 and 2020, Alaska census tracts were exposed on average to 0.2–2.9 *μ*g m^−3^ of smoke PM_2.5_ per day on an annual basis, with this range increasing to 0.6–7.0 *μ*g m^−3^ during the wildfire season, and to 3.5–49.8 *μ*g m^−3^ on the 10 smokiest days per year in each tract (figures 3(a)–(c)). Statewide, average daily wildfire smoke concentration was highest in the summer of 2004 and 2019 (8.4 *μ*g m^−3^ and 5.7 *μ*g m^−3^, respectively). In the 5 years when over 2 million acres burned in Alaska during the study period, between 86% and 98% of census tracts experienced at least 1 d of moderate smoke, and 19%–73% of tracts experienced dense smoke (figures 3(d) and (e)). Census tracts in the Fairbanks North Star Borough, Upper Yukon, and Tanana Flats experienced over 300 d of poor air quality due to wildfire smoke, across the study period (∼10% of summer days) (figures 3(d) and (e)). Most of Interior Alaska experienced 30–70 smoke waves. The longest smoke waves in this region lasted between 30 and 43 d in 2004, 2005, and 2009 and up to 20 d in Southcentral Alaska in 2014 and 2019 (figures 3(a)–(g)). Although historically, fire activity has been concentrated in Interior Alaska, communities throughout the state have been impacted by wildfire smoke (figure 3(h)).

Wildfire smoke metrics by year (supplemental figures 10(a)–(g)) show that exposure to wildfire smoke in Alaska varied substantially by wildfire season, and the spatial pattern of smoke often did not align with fire locations (supplemental figure 11). For example, in 2004, 2005, and 2009, at least a week of moderate or dense smoke days was observed in North Slope Borough, Northwest Arctic, and Nome census tracts despite no major fires recorded in those regions in those years.

### 3.2. Co-occurrence of high wildfire smoke exposure and high social vulnerability

**Figure 4.**
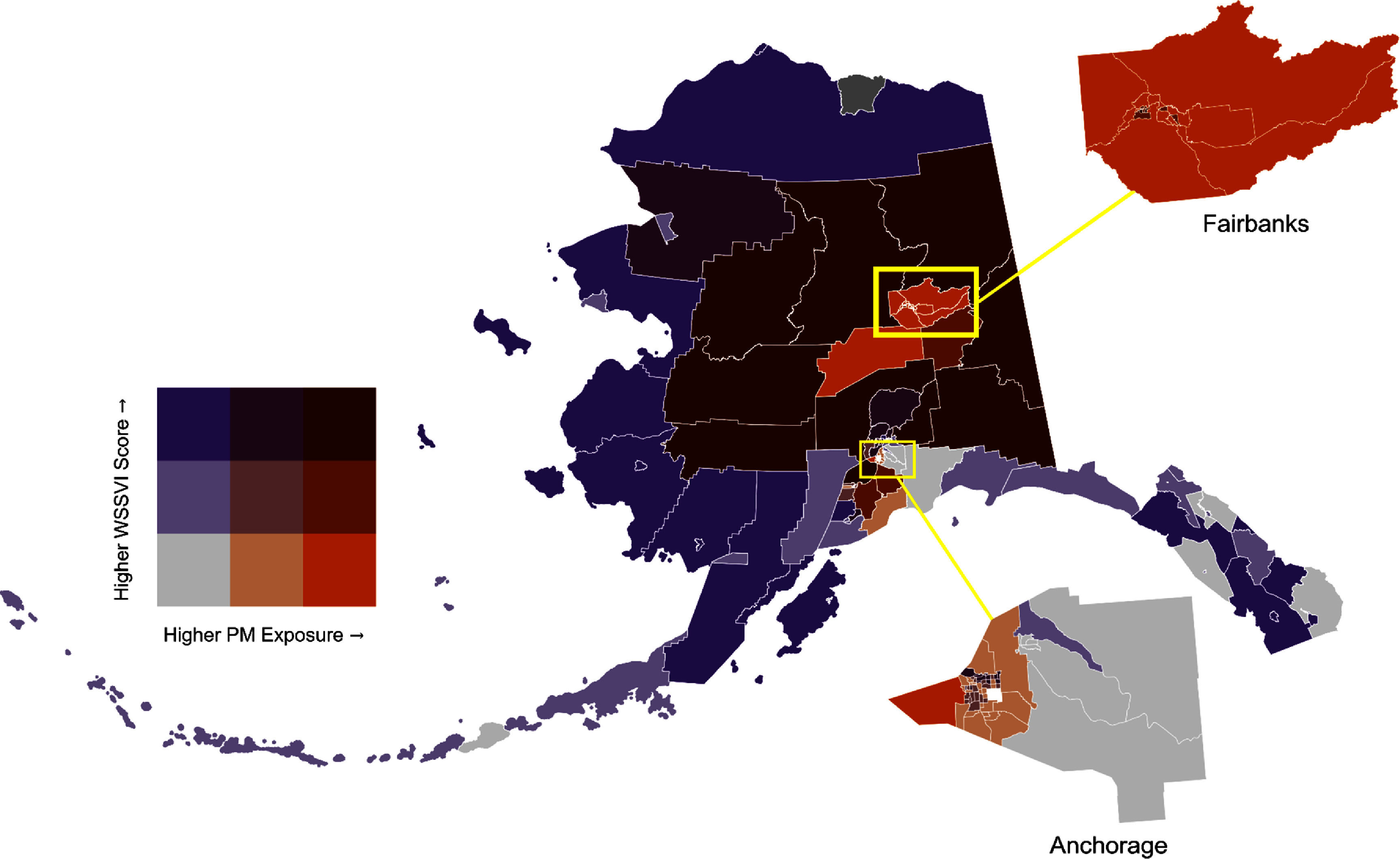
Wildfire-specific PM_2.5_ exposure and wildfire smoke social vulnerability index (WSSVI) rankings by census tract in Alaska, 2003–2020. Smoke exposure is defined as the number of days of moderate or dense smoke (⩾9.1 *μ*g m^−3^) in a census tract during the study period. WSSVI incorporates indicators of health sensitivity and adaptive capacity of the census tract population. Both variables are shown as percentile ranks where darker orange colors indicate higher smoke exposure, darker purple colors indicate higher smoke vulnerability, and a dark maroon color indicates both high smoke exposure and high smoke vulnerability.

There were 45 census tracts identified as having a dual burden of high smoke exposure and wildfire smoke vulnerability (figure 4). The majority of these tracts were located in rural areas in Interior Alaska; however, urban tracts in the population centers of Fairbanks, Anchorage, and the Kenai Peninsula were also identified in this high risk category. A quadrant map showing the smoke exposure and smoke vulnerability level of each census tract (supplemental figure 12) as well as maps of health sensitivity, adaptive capacity, and composite WSSVI rankings by census tract (supplemental figures 13(a)–(c)) are included in supplemental material to support planning efforts. Compared to low smoke exposure tracts, high exposure tracts had higher proportions of residents burdened by housing costs (24.8% vs 19.1%), individuals living below the 150% poverty line (19.1% vs 17.3%), women of childbearing age (22.6% vs 21.0%), and single-parent households (6.2% vs 5.4%) (supplemental table 3). All other WSSVI variables were lower in tracts with high historical smoke exposure. Only tract-level group differences between housing cost-burdened residents (*t* = 4.06, *p*= 0.001) and women of childbearing age (*t* = 2.822, *p* = 0.04) were found to be statistically significant.

## 4. Discussion

We estimated daily wildfire smoke PM_2.5_ at a 0.625° × 0.5° resolution across Alaska from 2003 to 2020, filling a critical gap in historical air quality for the region. Using this dataset, we developed smoke metrics to assess long-term and cumulative exposure and integrated these with social vulnerability indicators to identify Alaska communities disproportionately affected by smoke. While similar analyses have been conducted for California [25] and the contiguous US [41, 42, 81], our study advances air quality modeling, exposure assessment, and vulnerability assessment broadly while providing a comprehensive synthesis of these approaches for Alaska.

Our study builds on that of Chen *et al* [38] who performed a large ensemble of trajectory simulations to map daily smoke PM_2.5_ concentrations at a fine spatial resolution (1° × 1°) across Alaska from 2001 to 2015. Here, we used the 3D chemical model GEOS-Chem, which included background PM_2.5_ and a more accurate wet deposition scheme than that in HYSPLIT. Importantly, we relied on observational records to calibrate the modeled results with air quality data from AK DEC and PurpleAir sensors, thus eliminating bias at these sites, which tended to be close to more densely populated areas.

Traditional measures of wildfire severity, such as acres burned, fail to capture the true scale of human health impacts. As wildfire seasons grow longer and more intense, there is a need for metrics that measure not just fire extent but its consequences for people. We demonstrate that person-days of smoke exposure provides an alternative approach and provide several additional smoke metrics that can be used to quantify exposure over an entire wildfire season, or multiple years. Most wildfire smoke metrics were moderately to strongly correlated with one another (supplemental figure 14), although person-days of moderate and dense smoke exposure was only moderately correlated with the other metrics. This suggests that person-days of exposure captures a different aspect of population-level wildfire smoke exposure. Overall, these exposure measurement tools provide options for communicating with the public, enhance our understanding of long-term health risks of smoke exposure, and support public health planning, climate adaptation, and fire management, offering a more comprehensive approach as wildfire activity escalates globally.

We applied these metrics to Alaska and found that wildfire smoke is a chronic and widespread environmental hazard, to an extent that has likely been underappreciated. As in Chen *et al* [38], we found that the Interior region of Alaska has been most severely affected by smoke. For example, in 2004, most tracts in this region experienced annual wildfire smoke PM_2.5_ levels of 12–22 *μ*g m^−3^—approximately 1–1.8 times higher than the overall annual average PM_2.5_ from all sources across the US that year (12 *μ*g m^−3^) [82], and on par with or exceeding the highest annual smoke PM_2.5_ concentrations observed in the contiguous US in all but 1 year between 2007 and 2018 (also around 12 *μ*g m^−3^) [43]. During the 2004 wildfire season, the average daily smoke PM_2.5_ in this region was 35.8 *μ*g m^−3^, with some tracts reaching average daily levels >54 *μ*g m^−3^. In comparison, estimates of the contribution of wildfire smoke PM_2.5_ to summertime air pollution in the Pacific Northwest reached a maximum of 9.2 *μ*g m^−3^ in the most severe season in a 11 year study period [42]. During the 10 smokiest days per year in each census tract over our 18 year study, the 15% of the Alaska population living in the Interior region was exposed to wildfire smoke concentrations equivalent to smoking ∼1–2 cigarettes per day (22–50 *μ*g m^−3^ daily average) [83]. On these peak days in 2004, the wildfire smoke in census tracts in Fairbanks and areas immediately surrounding was equivalent to ∼5–17 cigarettes per day (120–370 *μ*g m^−3^ daily average) [83]. In California, estimates for wildfire smoke PM_2.5_ during the peak week of exposure reached a maximum of 31 *μ*g m^−3^ over a 15 year average, and up to 50 *μ*g m^−3^ for the year with the highest exposure [25]. Two factors may explain elevated smoke concentrations in Alaska: fire management practices and the unique characteristics of high-latitude fires. Due to Alaska’s vast size, fires in management areas classified as ‘limited’ priority for suppression are allowed to burn [84] if they do not threaten higher priority areas, which can lead to longer periods of smoke exposure. Additionally, high-latitude fires frequently burn in high-moisture duff layers, resulting in lower-temperature, smoldering combustion that produces more smoke compared to the hotter, faster-burning fires typical of lower latitudes [85, 86].

We based our wildfire smoke metric thresholds on the EPA AQI categories, which are commonly used to communicate health risks from ambient PM_2.5_ To define poor air quality from wildfire smoke, we used a threshold of 9 *μ*g m^−3^, corresponding to the lower end of the ‘moderate’ AQI range. This reflects growing evidence that wildfire-specific PM_2.5_ is more cytotoxic than ambient PM_2.5_ [87, 88], suggesting that health effects may occur at lower concentrations. Epidemiological studies have consistently identified increases in all-cause mortality, respiratory hospitalizations, and respiratory emergency department visits with increases in 1 *μ*g m^−3^ of smoke PM_2.5_ [13], supporting the use of more protective thresholds for wildfire-specific PM_2.5_.

During the study period, wildfire smoke resulted in poor air quality on 10% of summer days in some Alaska regions. Summer is a crucial time for outdoor activities that support physical and mental well-being, including recreation, subsistence food harvesting, and community events like culture camps and festivals. When poor air quality persists for a significant portion of the season, it can disrupt these activities, leading to indirect health impacts by limiting opportunities for exercise, social connection, and cultural practices [89, 90], all of which are essential for health and resilience. It can also disrupt economic livelihoods (e.g. tourism), causing additional mental burden [22, 90]. We also found that nearly every community in Alaska has experienced wildfire smoke, even though most wildfire activity is concentrated in the Interior. Wildfire smoke can travel hundreds of miles beyond the burned area [91–93], significantly degrading air quality in downwind regions. As a result, wildfires have become the sole driver of air quality exceedances in Alaska during summer, when other anthropogenic and natural sources are negligible. Smoke that travels from distant wildfires may impact health differently than smoke from nearby fires. People may take fewer precautions when there is no immediate fire threat, increasing their exposure, or transported smoke may have different chemical properties than local smoke, potentially affecting its toxicity [94, 95].

By combining a long-term wildfire smoke exposure metric with a measure of population susceptibility, we identified Alaska census tracts at the highest risk from wildfire smoke. The constructed WSSVI incorporates socio-demographic factors specifically linked to cardiorespiratory risks from smoke exposure, based on established research [14]. While the index may require updates as new risk factors emerge, it offers a more precise and relevant assessment of vulnerability to smoke-related health impacts compared to broader, all-purpose vulnerability indices [96, 97].

### 4.1. Implications for public health, climate adaptation, and wildfire management

One of the few publicly available wildfire smoke indicators is real-time AQI, which does not capture cumulative exposure. Metrics from this study, such as the number of days of poor air quality or person-days of smoke exposure, offer alternative, population-based ways to communicate the health risks of wildfire smoke. Presenting these easy-to-understand metrics alongside clear, actionable recommendations (e.g. staying indoors, using air filtration, or opening clean air centers) can encourage both individual behavior changes and community interventions [98]. Additionally, identifying vulnerable sub-populations in high-smoke areas (e.g. housing-cost burdened individuals, pregnant women) can inform targeted messaging that resonates with their lived experiences [99]. Research on wildfire smoke communication suggests that visual maps, messaging focused on protective actions, and diverse communication channels can enhance public understanding and engagement [98, 99].

During wildfire season, real-time AQI and fire location data are often used to assess conditions, but they do not fully capture population exposure or cumulative smoke impacts. Our metrics provide a more complete picture and can guide targeted responses, such as deploying air quality monitoring in high-exposure areas and directing medical response teams to at-risk populations. By integrating these metrics into emergency planning, response efforts can be more proactive and better aligned with the needs of affected communities.

Social vulnerability is widely used by emergency managers and planners to understand how social factors can increase or reduce the risks posed by environmental hazards [97, 100]. In this study, we applied social vulnerability mapping to assess the human impact of wildfire smoke in Alaska. By visualizing these results geographically and in a quadrant map, we provide a tool to support statewide climate adaptation and emergency management planning, helping to prioritize communities for investments in fuels reduction and smoke resilient housing and community buildings. With the integration of real-time cumulative wildfire smoke exposure data, this resource could also support decision-making during active wildfire seasons.

The increasing severity of wildfires in the western US and other fire-prone regions [39, 101] is driving innovation and the need for cross-disciplinary collaboration in wildfire management, with a growing emphasis on public health and environmental justice [23, 28]. Prescribed burns and revitalization of cultural burning, for example, are gaining recognition as forest management tools that can reduce overall smoke exposure by controlling when and where fires occur [102, 103]. Current US regulations governing prescribed fires focus primarily on air quality standards and do not account for the vulnerability of nearby populations [23, 47]. Our integrated wildfire smoke and social vulnerability assessment offers a framework for addressing this gap. Additionally, our wildfire smoke metrics provide a practical way to incorporate public health considerations into forest management by offering a shared language to evaluate the health risks of different fire management strategies.

We acknowledge several key limitations in our assessment. Our GEOS-Chem nested simulation was performed for nearly two decades at a horizontal resolution of approximately 69 × 55 km, comparable to other global models. We anticipate that regional models or satellite retrievals with finer resolutions (10 km or less) will further improve the representation of spatial variability, although they may face challenges related to computational demands or retrieval accuracy [104]. The GEOS-Chem model sometimes produces low PM_2.5_ concentrations (<5 *μ*g m^−3^) over broad areas far downwind of wildfires (supplemental figure 15). This tendency likely reflects smoke diffusion, which can sometimes be unrealistic in coarse-grid Eulerian models like GEOS-Chem that cannot capture the fine spatial structure of smoke plumes [105]. It could also arise from formation of secondary aerosols resulting from plume aging. Whatever the causes of these minor PM_2.5_ enhancements, we focused here on smoke concentrations >9 *μ*g m^−3^ for our metrics. While our percentile-mapping method yielded reasonable PM_2.5_ estimates, there is room for improvement given the overall *R*^2^ of 0.60. Another limitation of our approach is our relatively simple method of redistributing smoke emissions through the PBL in GEOS-Chem, which may not reflect the complexity in plume injection heights as shown from observations [34]. And given our model setup, we are unable to distinguish locally sourced smoke from smoke that has traveled long distances. In addition, while the variables included in the WSSVI were selected based on empirical evidence from published studies, they do not fully capture all aspects of a community’s capacity to respond to wildfire smoke. And because our analysis is based on census tract-level data, which represents averaged household characteristics, it may not reflect the full range of vulnerability within each tract.

## 5. Conclusion

Wildfire smoke is a persistent and growing public health challenge. This study highlights the need to move beyond traditional fire metrics and adopt measures that better capture the full scope of human exposure. By quantifying cumulative smoke exposure and integrating social vulnerability, we provide tools that can be used to guide public health responses, inform policy, and prioritize resources for those most at risk. The wildfire smoke metrics developed here are broadly applicable across fire-prone regions. They offer a way to improve risk communication, ensure emergency planning accounts for population susceptibility, and integrate public health into wildfire management decisions. As wildfires continue to intensify, these approaches can help shift smoke response from reactive to proactive and enable communities to better prepare, adapt, and protect public health in an era of increasingly smoky summers.

## Data Availability

The data that support the findings of this study are openly available at the following URL/DOI: https://github.com/Hahn-Research-Group/Alaska_wildfire-smoke-metrics_2025.
